# Supplementary material for: Application of big-data for epidemiological studies of refractive error
Source: PLoS One. 2021 Apr 23;16(4):e0250468. doi: 10.1371/journal.pone.0250468 (PMC8064549; doi:10.1371/journal.pone.0250468)
Supplement: Attachment — Submitted filename: Response to Reviewers.docx [file pone.0250468.s001.docx]

Michael Moore
Centre for Eye Research Ireland
School of Physics & Clinical & Optometric Sciences

Technological University Dublin

Dublin

Ireland

Email: [michael.moore@tudublin.ie](mailto:michael.moore@tudublin.ie)

Michael Mimouni, MD

Academic Editor

PLOS ONE

23/02/2021

Dear Dr. Mimouni,

Thank you for requesting us to submit a revised draft of our manuscript entitled “Application of Big-Data for Epidemiological Studies of Refractive Error” to PLOS ONE. We sincerely appreciate the time and effort undertaken by you and each of the reviewers when considering our manuscript and in providing your feedback. We are delighted to resubmit our manuscript for further consideration having incorporated changes that reflect the comments you have provided.

To facilitate your review of our revisions, the following is a point-by-point response to the questions and comments delivered in your email of 22/02/2021.

**Editor Suggestions:**

1. Please ensure that your manuscript meets PLOS ONE's style requirements, including those for file naming

***Thank you for your feedback. We have made edits to the title page and manuscript to ensure they conform to PLOS ONE’s style requirements***

2. Please provide an amended Funding Statement declaring this commercial affiliation, as well as a statement regarding the Role of Funders in your study

***This has been updated on the submission portal and in the accompanying cover letter***

3. Please also provide an updated Competing Interests Statement declaring this commercial affiliation along with any other relevant declarations relating to employment, consultancy, patents, products in development, or marketed products, etc.

***This has been updated on the submission portal and in the accompanying cover letter***

4. We note that you have indicated that data from this study are available upon request. PLOS only allows data to be available upon request if there are legal or ethical restrictions on sharing data publicly. For information on unacceptable data access restrictions, please see http://journals.plos.org/plosone/s/data-availability#loc-unacceptable-data-access-restrictions.

In your revised cover letter, please address the following prompts:

a) If there are ethical or legal restrictions on sharing a de-identified data set, please explain them in detail (e.g., data contain potentially identifying or sensitive patient information) and who has imposed them (e.g., an ethics committee). Please also provide contact information for a data access committee, ethics committee, or other institutional body to which data requests may be sent.

b) If there are no restrictions, please upload the minimal anonymized data set necessary to replicate your study findings as either Supporting Information files or to a stable, public repository and provide us with the relevant URLs, DOIs, or accession numbers. Please see http://www.bmj.com/content/340/bmj.c181.long for guidelines on how to de-identify and prepare clinical data for publication. For a list of acceptable repositories, please see http://journals.plos.org/plosone/s/data-availability#loc-recommended-repositories.

***This has been updated in the accompanying cover letter***

**Reviewer 1 comments:**

1. Limitations of the study are generally well considered; however, I feel that some of the statements related to the use of the EMR data should be made more balanced. The authors should carefully consider the limitations of using the EMR data to obtain a measure of the prevalence and distribution of refractive errors in the population. I somewhat disagree with the statement that “Undersampling of emmetropic patients is likely to be less problematic for the EMR data which includes refraction data found as part of a patient’s eye examination.” The undersampling of emmetropes is likely to be quite prominent in young adults who are unlikely to visit an optometry practice unless they have myopia. Interestingly, EMR dataset shows higher prevalence of myopia among young individuals compared to E3. I was left with the impression that I could not decide whether this was a result of a genuinely higher prevalence of myopia in Ireland compared to the overall European sample, or this was a result of a bias cause by the undersampling of emmetropes.

***Thank you for your feedback. We have edited this point to take the reviewers comment into account:***

***“There are less limitations applicable to the EMR data due to the increased demographic detail captured in this data. Under sampling of young emmetropic patients may still present an issue for the EMR data which includes refraction data found as part of a patient’s eye examination. Young patients without significant refractive error are less likely to attend an optometry practice and this may explain the higher levels of myopia observed in young age groups when compared to the E3 data (Fig 7). Despite this, EMR data is still likely more representative than spectacle lens data for young patients as some will still attend for the purposes vision screening for occupational requirements and driving licensure and for screening of common ocular pathologies such as glaucoma and cataract [46].”***

2. Both EMR and E3 datasets show sharp increase in the prevalence of myopia in younger generations 25-29 compared to other age groups, which is important to emphasize.

***We agree this point should be emphasised and have added the following point in the first paragraph of the discussion:***

***“Both the EMR and E3 datasets demonstrated high levels of myopia in younger age groups (Fig 7) which supports the findings of other studies demonstrating an increase in myopia prevalence in more recent generations [5,6].”***

***As mentioned in point 1 above we have also more clearly indicated there is some uncertainty with this finding later in the discussion:***

***“Young patients without significant refractive error are less likely to attend an optometry practice and this may explain the higher levels of myopia observed in young age groups when compared to the E3 data (Fig 7).”***

3. Please, clearly identify in the text and figure/table legends what dataset is being analyzed/discussed.

***The text and figure/table legends have been edited to more clearly identify the dataset being discussed***

Thank you for giving us the opportunity to improve our work through your valuable feedback. We have tried to incorporate your comments and hope these revisions will encourage you to accept our submission.

Yours Sincerely,

Michael Moore
